# 

**Published:** 1884-04

**Authors:** 


### NEW BOOKS, ETC.

MEDICAL INDEX.—The Burr Index Company, of Hartford, Conn., have prepared a blank book, of ample dimensions, suitably and very conveniently lettered on the margins, by means of which the physician, given to considerable reading, can index all medical themes in which he is especially interested, The marginal letters are stamped upon leather, permitting of any amount of thumbing, while the various combinations of letters under a given head makes the finding of any subject very easy. The book is well bound, of extra heavy paper, and is admirably gdapted to the wants of the medical man. The index to the BISTOURY in the last number was prepared through its aid, and made the labor very easy and accurate.

---

NEW BOOKS RECEIVED.—COPP'S U. S. SALARY LIST AND CIVIL SERVICE RULES.—Our many readers will welcome the solid information contained in the 160 pages of this recently-issued book. It is prepared by Henry N. Copp, a lawyer of Washington, D. C. All the government salaries are given from President Arther's $50,000 to postmasters with $500, officials of the Treasury, Interior, War and Navy Departments, Custom Houses, post offices, and fully 20,000 federal offices arranged by States and Territories. Specimen examination questions for admittance to the Civil Service throughout the country are added. The price of the book is only 35 cents.

---

FISHING WITH THE FLY.—This is a book that will at once captivate followers of the gentle art. It contains sketches from all the prominent lovers of fly fishing who have been in the habit of writing upon this entertaing theme. The book is compiled by Charles F. Orvis, who contributes a number of full-page illustrations of various flies most beautifully colored—the very sight of which causes the angler's dorsal fin to vibrate, and A. Nelson Cheney, who feelingly writes of the delights of angling.

We heartily recommend the book to all lovers of the rod.

To be had of C. F Orvis, Manchester, Vt.

---

THE SCIENCE MONTHLY—A monthly journal of Notes and News in all departments of science. Published by S. E. Cassino & Co., 41 Arch street, Boston, Mass. $1.00 a year.

This is an excellent little journal containing much of interest to workers in all departments of scientific research. While not aiming to take the place of more ambitious periodicals devoted to special branches of investigation, it furnishes an admirable monthly review of scientific progress, and affords a remarkable amount of condensed information to the busy worker who, while devoted to some special study, yet wishes to keep posted upon the general advance in science in all departments.

